# What are the basic self-monitoring components for cardiovascular risk management?

**DOI:** 10.1186/1471-2288-10-105

**Published:** 2010-11-12

**Authors:** Alison M Ward, Carl Heneghan, Rafael Perera, Dan Lasserson, David Nunan, David Mant, Paul Glasziou

**Affiliations:** 1 Department of Primary Health Care, The University of Oxford, Old Road Campus, Headington, OX3 7LF, UK

## Abstract

**Background:**

Self-monitoring is increasingly recommended as a method of managing cardiovascular disease. However, the design, implementation and reproducibility of the self-monitoring interventions appear to vary considerably. We examined the interventions included in systematic reviews of self-monitoring for four clinical problems that increase cardiovascular disease risk.

**Methods:**

We searched Medline and Cochrane databases for systematic reviews of self-monitoring for: heart failure, oral anticoagulation therapy, hypertension and type 2 diabetes. We extracted data using a pre-specified template for the identifiable components of the interventions for each disease. Data was also extracted on the theoretical basis of the education provided, the rationale given for the self-monitoring regime adopted and the compliance with the self-monitoring regime by the patients.

**Results:**

From 52 randomized controlled trials (10,388 patients) we identified four main components in self-monitoring interventions: education, self-measurement, adjustment/adherence and contact with health professionals. Considerable variation in these components occurred across trials and conditions, and often components were poorly described. Few trials gave evidence-based rationales for the components included and self-measurement regimes adopted.

**Conclusions:**

The components of self-monitoring interventions are not well defined despite current guidelines for self-monitoring in cardiovascular disease management. Few trials gave evidence-based rationales for the components included and self-measurement regimes adopted. We propose a checklist of factors to be considered in the design of self-monitoring interventions which may aid in the provision of an evidence-based rationale for each component as well as increase the reproducibility of effective interventions for clinicians and researchers.

## Background

As cardiovascular disease (CVD) is still the leading cause of death worldwide, better methods are needed to manage CVD risk[1]. One increasingly common method is self-monitoring to improve the adjustment and effectiveness of long-term treatments,[2-5] and guidelines indicate self-monitoring in several CVD areas. Heart failure (HF) patients can self-monitor weight and adjust therapy in response to treatment [6]. Patients with atrial fibrillation requiring anticoagulation can self-monitor their INR levels, [7,8] and those with high blood pressure (BP) can self-monitor at home[9]. However, not all self-monitoring is effective: self-monitoring of blood glucose in non insulin treated diabetics is also recommended[10] but recently randomised trials have questioned its value[11]. The effectiveness of self-monitoring reported in different clinical trials varies substantially. Even a cursory scan of these trials shows that this variation might be explained at least in part by the substantial variation in how the self-monitoring was done in each trial.

Self-monitoring has long been part of behaviour change in the psychological field where it was used in a more subjective self-assessment mode [12]. To be effective in the medical context three minimum criteria are required: firstly, clinically significant changes in the condition are possible over time; secondly, an objective test exists that reliably detects these changes; and finally a cost effective action in response to the test result is possible[13].

One of the early examples of self-monitoring was an asthma programme developed for children [14,15]. This programme was informed by the early work on self-efficacy by Bandura [16] and included the patient as an active participant in the management of their illness. Self-monitoring is itself a complex intervention made up of a number of components which can act both independently and interdependently[17-19]. The necessity and relative value of these individual components is poorly understood, even though understanding how complex interventions work is important for effective implementation[20]. Yet in CVD these complex interventions have been widely adopted with little understanding of their active component parts. Therefore to better understand the components that make up self-monitoring interventions for CVD from randomised trials we chose to analyse trials in oral anticoagulation, hypertension, blood glucose in type 2 diabetes and heart failure.

## Methods

We selected published systematic reviews that evaluated the effects of self-monitoring of CVD in four specific areas: 1) oral anticoagulation 2) hypertension, 3) diabetes and 4) heart failure. We searched MEDLINE (1950 to Dec 2008) using a combination of MeSH terms and free-text keywords, limiting our searches to systematic reviews using a HIRU hedge for systematic reviews. The Cochrane Database of Systematic Reviews (CDSR) and the Database of Abstracts of Reviews of Effectiveness (DARE) were searched using the same terms through the Cochrane Library (2008, Issue 4). We used a cascade approach to locate further reviews by hand searching retrieved articles and citation searches of these articles. Inclusion criteria for each disease were: the most recent systematic review of randomized controlled trials of self-monitoring interventions. We then retrieved the full text published randomized controlled trials (RCTs) cited in the systematic reviews.

In a previous study we developed a template to assess whether a clinician could use the treatment described in a randomized trial with a patient tomorrow [21]. Based on this template three of the authors (PG, AW, CH) designed extraction tables which included all the elements of self-monitoring interventions which we considered would be needed to be able to reproduce an intervention. We divided these into two tables: one for all the elements related to education and training and one for all elements of monitoring. We piloted these extraction tables within the anticoagulation RCTs. We then added a third table which covered the theoretical basis of the education provided, the rationale given for the self-measurement regime adopted and the compliance with the regime by the patients. We then extracted the components for each disease using the three data extraction tables. The data extracted for the education component were: delivery, number, length, mode, content and assessment of training sessions. The data extracted for the monitoring component were: interval between measurements, recording of measurements, what is adjusted and by whom, adjustment algorithm, contact with health professionals, when to contact clinic, quality control of home measurement and transmission of data to study coordinators. All data extraction was checked by a second author and any discrepancies resolved by consensus.

## Results

In total we extracted data from 52 RCTs (10,388 patients): 14 HF trials (4,264 patients); 14 OAT trials (3,049 patients); 18 BP trials (1,714 patients) and 6 SMBG trials (1,361 patients). Remote monitoring in heart failure (HF) is effective in reducing all cause mortality (Relative Risk = 0.62 (95%CI 0.45 to 0.85) [2]. Self-monitoring of INR for patients on oral anticoagulation therapy leads to fewer thromboembolic events (Odds Ratio = 0.27 (0.12 to 0.59)), and lower mortality (OR = 0.37 (0.16 to 0.85)) [3]. Self-monitoring of blood pressure leads to reductions in systolic BP of 4.2 mmHg (95% CI, 1.5 to 6.9) as well as diastolic pressure 2.4 mmHg (95% CI, 1.2 to 3.5) [4]. Self-monitoring of blood glucose (SMBG) which has been found to be effective for patients with type 1 diabetes and type 2 diabetes if they are taking insulin appears not be effective in improving HbA1c in with patients with type 2 diabetes using oral hypoglycaemic drugs [5].

### Components of self-monitoring

We have summarised the data extracted into four main component areas forming the self-monitoring interventions described in the trials: a) education b) self-measurement c) adjustment of (or adherence to) medication and/or behaviour d) contact with health professionals. Not all components were evident in every trial, and in some cases the intervention was too poorly described to be clear whether or not a component was present.

#### 1) Education

The first component in a self-monitoring intervention is patient (and sometimes health professional) education. At its most basic, once patients have been identified as suitable for self-monitoring they need to be taught how to use the self-monitoring equipment [22]. However, the education can also be used to provide patients with information on disease, management and lifestyle [23]. Initial training may be necessary for the health professionals involved [24] to deliver an effective education session. A theoretical basis for the type of training provided has also been suggested [25,26].

The intensity and structure of education varied considerably even for the same clinical problem. In all the heart failure trials, provision of education was part of the interventions with ongoing phone contact by study staff for counselling and education in addition to the monitoring of signs and symptoms. Nonetheless, the amount of education varied. One study [22] did not mention education about heart failure or counselling during monthly phone calls at all and only reported education on the operation of the telemonitoring equipment. In contrast another trial provided one-to-one or group counselling covering disease, management, lifestyle and monitoring as well as providing extensive educational materials. The patients were also assessed before and after each session and were able to contact medical staff for advice and help [23]. No trials mentioned any theoretical basis for their educational strategies.

In self-monitoring of INR studies, most trials gave two to three educational sessions, including an assessment of competency. One trial [27] based their educational component on Social Learning theory [16,28] and many trials referred to each other to determine the education strategy [29-32].

In BP self-monitoring trials, education varied from very comprehensive (education provided to both the patients and health care providers)[24] to patients simply being instructed in the use of the monitor[25]. Training and education was based on a theoretical model in only one trial [26]; the model adopted was the Health Belief Model [33].

For the self-monitoring of blood glucose trials, education ranged from ongoing counselling and education using an algorithm delivered monthly by a trained nurse[34] to instructions in the use of a monitor and renewal of dietary recommendations twice [35]. Again no theoretical basis was given for delivery of the education provided, though one study used behavioural rather than didactic education as a means of increasing compliance [36].

#### 2) Self-measurement

The obvious defining feature of self-monitoring is self-measurement. Hence a major focus of the initial education was on the "how to" and the interpretation of self-measurements. Self-measuring regimes have to take into account the accuracy of the monitoring device, the run in period required to ensure patients are safe and effective at self-measuring, the quality assurance of the monitoring device and the frequency with which patients are required to self-measure.

Ignoring the evidence underpinning self-measurement regimes led to poor ongoing research. For example, in an early trial of blood pressure self-monitoring, daily self-measurement of blood pressure proved too much for most patients [37] with only 1/5^th ^of patients allocated to the intervention completing the trial. Yet 11 of the 18 hypertension trials asked the patients to measure daily or more frequently without any rationale given for doing so [4].

Considerable variation occurred in what patients were asked to monitor and the extent of external monitoring in the trials. The minimum heart failure patients were asked to do was measure their weight, record their medications daily and monitoring by a monthly phone call by a research coordinator [38]. In contrast, other patients were asked to electronically measure weight, blood pressure, heart rate and rhythm twice a day and transmit results immediately to the study centre[22].

In the INR trials, only one study mentioned a rationale for the frequency of testing. This trial suggested testing twice a week was optimal to keep patients within the target therapeutic range based on previous research [39]. There was considerable variation between what patients were told to do and what they actually did in terms of frequency of INR measurements. For example, in one trial they were instructed to take 11 measures over the 6 months and whilst the median number of measures performed was 17, the range was 2 to 39 [27]. Only 6 trials (43%) mentioned external quality control of the tests [29,30,40-43].

In the BP trials, self-measurement varied considerably as well as the mode of recording and the responses to the readings (self-management or other). This ranged from patients taking electronic BP self-measures three times in the morning and evening at least three times a week with the readings being automatically transmitted to the study centre, providing patients and physicians with weekly averages, but compliance with this regime was not reported [44]. In contrast, in another trial patients were asked to take two consecutive readings twice a week and mail results to the study centre once a month but only 50% of patients were compliant[45]. No trial gave a rationale for the monitoring regime the patients were asked to perform which ranged between 1[46] and 21[47] blood pressure readings per week. Overall compliance with the measuring strategies ranged from 15% to above 90%.

The trials of self-monitoring of blood glucose in patients with type-2 diabetes varied in the number of tests patients performed and in whether tests were pre or post-prandial. Whilst some authors specifically hypothesised that postprandial changes in glucose were important [34,35,48,49], none gave any rationale for their decision on how often patients were asked to test which ranged from 24 [50] to 48 [34,48] times per month. Reported compliance with testing ranged from 45% to above 90%, however, in some cases patients tested twice as often as requested, 25 times a week instead of 12 [34]. Quality assurance of measurements was mentioned in only two of the six trials [34,50].

### Adjustment/Adherence

In self-monitoring some subsequent action needs to occur to lead to a clinical change: either adjustment of treatment or better adherence to treatment. If the purpose of the self-monitoring is to increase motivation or reinforce behaviour then there appears to be no point in re-testing before the behaviour has had time to produce a meaningful change in what is being measured. For example, the lack of effective blood glucose self-monitoring [5] where the results of behaviour changes to diet and exercise do not have time to effect what is being monitored. An example of where this does work well is self-management of INR where the test results inform medication dose adjustments, the effects of which can be seen in the next test result [3]. The relatively small effect size found in the blood pressure trials may also be due to the fact that the patients do not make adjustments in response to their test results or their tests are too frequent for meaningful changes to have taken place.

The heart failure trials differed in their aims and consequently in what the patients were asked to do. In more intense interventions the aim was to provide clinicians with diagnostic information once or twice daily to improve titration of medications [22]. In direct contrast other trials were designed to do less self monitoring and more self-care, while adjustments of therapy were minimal[38]. Patients carried out their own dose adjustments in two-thirds (64%) of the INR trials but in only one of the blood pressure trials [51] where adherence to medication was more often the purpose of the self-monitoring rather than adjustment of therapy. In the diabetes trials the patient self-adjustments were to lifestyle and nutrition with any medication adjustments being carried out by an external health professional [35,36,48,50].

### Health care professionals

It is clear that the purpose of the contact with health professionals in many of the trials was to increase compliance with medication and measurement and provide physicians with information for therapy adjustment. Contact with health professionals was also a way of periodically reinforcing or updating education. However, it is not clear how much contact is optimal and whether contact is best by phone, home visit, clinic visit or computer.

In most of the heart failure trials the purpose of the contact was mainly for monitoring symptoms, medication, adherence and education and advice. The health professionals contacting the patients were mainly nurses with only two trials using pharmacists[52,53] and one a research co-ordinator[38]. The mode of contact was phone in all but two trials where videoconferencing was used[54,55]. The amount of contact varied from three calls by a pharmacist over 6 months [52] to 17 calls from a nurse (median 14 range 11 to 22) over 6 months[56].

In the INR trials the ongoing contact was mainly for safety. Contact was either with clinicians or nurses and in one study a pharmacist also contacted patients monthly [42]. Some patients were able to contact a 24 hour help desk,[29]or a clinician available 24 hours [27] or during work hours [40] though in most trials patients were given instructions on when they needed to contact a health professional.

In the BP trials the purpose of the contact with health professionals varied from motivation[57] to medication adjustment during phone calls or clinic visits[44]. Most of the contacts were with doctors or nurses during clinic visits but in one case the contact was with a study co-ordinator who had no health professional training[57], in another a pharmacist contacted the patients[58] and in one the contact was with a telephone linked computer system[46]. The frequency of contact varied from mean of 1.5 clinic visits (SE 0.1) over 12 months[59] to weekly phone calls from a nurse for counselling[26].

The purpose of the contact with health professionals in the blood glucose trials was both for education and motivation. Dieticians[35,48,49], doctors and nurses [34,36,49,50]contacted the patients. The trials were 24 to 28 weeks with five[35] to at least 15[49] contacts over that period.

Finally, few trials mention a theoretical basis for the mediating effect of the results of the self-measurement on the patients' behaviour. For example, what is the theoretical basis for the assumption that the blood pressure self-monitoring results will increase compliance with hypertension medication?

Based on the gaps identified we summarised the factors to be considered at each stage in the design of a self-monitoring intervention in table 1. These factors cover aspects related to purpose, people, content and timing of the four main components of self-monitoring interventions. Potentially these factors could be used as a checklist when designing self-monitoring interventions for CVD disease (table 1). Answering the relevant questions in the table could not only improve the evidence base of the interventions but may also make them more reproducible.

**Table 1:** Self-monitoring components - factors to be considered at each stage in the design of a self-monitoring intervention

Stage				
**Factor**	**Education**	**Self-measurement**	**Adjustment/Adherence**	**Health care professional contact**

**Purpose**	*What is the purpose?* • To increase knowledge • To provide skills • To increase compliance • To increase motivation	*What is the purpose?* • To provide information • To increase compliance • To increase motivation	*What is the purpose?* • Titration of medication • Titration of behaviour • Adherence to medication regime • Adherence to behaviour regime	*What is the purpose?* • To provide education • To increase compliance • To increase motivation • Safety

	

**People**	*Who receives the education?* • Patients • Health care providers	*Who is the information for?* • Patients • Health care providers	*Who adjusts/adheres?* • Patients • Health care provider	*Which health professionals?* • Doctors • Nurses • Other

	

**Content**	*What type of education?* • Theoretical basis • Content • Mode of delivery • Support materials	*What test is to be used?* • Accuracy of the test • Feasibility in this setting	*What is adjusted/adhered to?* • Medication • Behaviour	*What is the format of the contact?* • Effectiveness • Cost

	

**Timing**	*What timing is optimal?* • Should it be once off or repeated • How long between sessions • Is it sustainable	*What timing is optimal?* • How long should the run in be *What is the frequency of measurements and does it take account of:* • The signal to noise ratio • Fatigue factor and compliance	*What is the frequency of adjustments considering:* • The signal to noise ratio • Fatigue factor and compliance	*What is the timing of the contact?* • Feasibility • Compliance

	

**Other**	*Should the learning be assessed?* • How many assessments • What level of success before allowing self-measurement • How much re-training	*What quality assurance is required?* • Internal QA and External QA • How often should QA be conducted? *What is recorded?* • How reliable is the recording method • How accurate is the recording method • Is electronic recording available and feasible	*What guidance is provided?* • Algorithm • Web based guidance • Clinician guidance	*Other?* • Algorithm based contact • Video conference

## Discussion

Analyses of CVD risk management by self-monitoring shows considerable variation in the components of the interventions between trials for the same disease. To what extent these factors impinge on results is difficult to determine, not only because of the lack of separate outcomes for individual components but also because the components of an intervention are often poorly described. In part this will be due to pressures of word counts in publications of reports [21,60] but it may also be due to the lack of consideration of the implications of the decisions made about each component in their design.

Clinicians use self-monitoring for CVD patients for different reasons. Blood pressure self-monitoring has been mainly used to improve adherence to medication [57] with patients in only one out of 18 trials adjusting their own therapy[51]. In contrast self-monitoring of INR in patients on oral anticoagulant therapy is used for titration of medication either by the patient or the physician [3]. Self-monitoring in heart failure patients can be to motivate the patient to improve diet and fluid intake, adhere to medications and also to provide clinicians with information on which to take preventive action to reduce hospital readmissions [2]. The various components of the self-monitoring will vary according to the purpose of the self-monitoring.

Using self-measurement alone to improve adherence appears to be less successful than when the patient can make some adjustment in direct response to the test result. Patients carried out their own dose adjustments in two-thirds (64%) of the INR trials with a considerable reduction in mortality OR 0.37 (0.16 to 0.85) [3] but in only one of the blood pressure trials [51] where the effect size was much less [4]..

The need for evidence-based rationales for the choice of each component is demonstrated by the poor compliance with many of the self-monitoring regimes adopted and lack of efficacy and minimal impact in some of the trials. Breaking self-monitoring into its component parts allows analysis and optimisation of each component. The rationale for each component needs to be explicit in both the design and reporting of research interventions and the translation of the interventions into clinical practice. Table 1 expands each component by providing a checklist of factors to be considered. In the long term what is sufficient for each component needs to be established by the relevant expert groups for each disease. In the meantime, this checklist can provide an aid to clinicians and researchers in the design and reporting of self-monitoring interventions for CVD risk management.

The present overview is limited by only having access to the published data. This means important components of the interventions may not have been reported mainly due to space limitations. In addition, our components were only based on trials included within the systematic reviews. Consequently, we may have missed important trials published subsequently that may have additional elements which would add to the components. Methods that improve the descriptions of complex interventions need to be developed. Previous work has highlighted the importance of better descriptions complimenting existing systematic review methodology[21,61].

## Conclusions

We propose that self-monitoring for CVD risk management can be broken into four major components: education, measurement, adjustment/adherence and contact with health professionals. These components are underpinned by a theoretical basis for the mediating effect of the self-measurement results on behaviour (adjustment and/or adherence). We have examined the latest systematic reviews of self-monitoring interventions for CVD management and found considerable variation in these components as well as a lack of evidenced-based rationales for their content. We have proposed a suggested checklist of factors to be considered at each stage in the design of self-monitoring interventions which may aid in the provision of an evidence-based rationale for each component as well as increase the reproducibility of effective interventions for clinicians and researchers. Despite current guidelines for self-monitoring in CVD management we suggest that for self-monitoring to be effective in practice all components of self-monitoring need to be considered.

## Authors' contributions

AW and CH conceptualized the study. AW, CH, DL, RP, DN conducted literature searches, extracted the data and summarised the results. AW and CH drafted the manuscript. All authors commented and revised the manuscript.

## Pre-publication history

The pre-publication history for this paper can be accessed here:

http://www.biomedcentral.com/1471-2288/10/105/prepub
